# Bioceramics Enhance the Anti-Tumor Activity of Immune Cells in Adoptive Immunotherapy

**DOI:** 10.3390/ijms251910567

**Published:** 2024-09-30

**Authors:** Masato Nose, Aiko Nitta, Yundi Zheng, Rihoko Kizukuri, Yuki Nagao, Shigenori Nagai, Mamoru Aizawa

**Affiliations:** 1 Applied Chemistry Program, Graduate School of Science and Technology, Meiji University, Kawasaki 214-8571, Japan; nooose4156@gmail.com (M.N.); aiko.sagittarius@gmail.com (A.N.); digong990213@gmail.com (Y.Z.); rkizukuri@gmail.com (R.K.); ficedula-narcissina@yahoo.ne.jp (Y.N.); 2 Department of Molecular Immunology, School of Medical and Dental Sciences, Tokyo Dental and Medical University, Bunkyo-ku, Tokyo 113-8945, Japan; nagai.mim@tmd.ac.jp; 3 Department of Oral Biology, School of Medical and Dental Sciences, Institute of Science Tokyo, Tokyo 153-8505, Japan; 4 Meiji University International Institute for Materials with Life Functions, Meiji University, Kawasaki 214-8571, Japan

**Keywords:** boron-containing apatite, adoptive immunotherapy, anti-tumor effects, immune cells, sol–gel process

## Abstract

Recent research has focused on immunotherapy with no side effects as an innovative medical treatment for cancer. However, typical drugs for immunotherapy are very expensive. Here, we propose the use of immunoceramics that activate immune cells by contact with their surface. Previous studies demonstrated that polymers, including the phenylboronic acid group, could activate lymphocytes. This activation may be due to the interaction between the sugar chains in cells and the OH group in B(OH)_3_ formed via the dissociation of the BO_2_ group. We have clarified that boron-containing apatite (BAp) activated lymphocytes in vitro. In this study, we fabricated the ceramic surfaces using the CaO-P_2_O_5_-SiO_2_-B_2_O_3_ system (CPSB ceramics) containing BAp as a main crystalline phase. The results of the in vitro evaluation indicated that killer T cells in splenocytes cocultured with the CPSB ceramics were more numerous than in splenocytes cocultured on a control surface. The results of the in vivo evaluation indicated that the CPSB ceramics significantly inhibited tumor growth when CD8-positive T cells were cultured on individual ceramics and subsequently injected into tumor-bearing mice. The present CPSB ceramics are expected to be a valuable biomaterial for immunotherapy.

## 1. Introduction

Cancer is a leading cause of death before the age of 70 in 112 out of 183 countries (World Health Organization, 2019). An estimated 19.3 million new cancer cases and almost 10.0 million cancer deaths occurred worldwide in 2020 [1]. Therefore, cancer is a major challenge that must be solved. The three main cancer treatment methods are (i) surgical therapy, (ii) radiation therapy, and (iii) chemotherapy. However, these methods present risks and side effects for patients and a declining quality of life. Immunotherapy, which triggers almost no side effects, is expected to become the fourth cancer treatment method [2]. With immunotherapy, activated immune cells attack tumor cells and induce apoptosis [3]. It enhances the patient’s immune system to strengthen its ability to attack cancer and is expected to reduce side effects.

Among attempts to use immunotherapy as a novel cancer treatment, two main approaches have been considered: adoptive immunotherapy and immune checkpoint therapy. This study focuses on adoptive immunotherapy, a treatment in which immune cells that lead tumor cells to apoptosis are first activated outside the body and then returned to the patient’s body. Following that approach, Rosenberg et al. developed lymphokine-activated killer (LAK) therapy using patients’ lymphocytes [4]. They reported that naturally occurring tumor-reactive lymphocytes contributed to the complete regression of melanoma. However, LAK therapy presents two challenges: its low specificity for cancer antigens and the use of large amounts of IL-2 in the lymphocyte culture. Robbins et al. developed a genetically modified T cell receptor (TCR) therapy in which a T cell receptor gene that recognizes cancer antigens is transfected into the patient’s T cells and infused with T cells with increased specificity for the cancer antigen [5]. The results indicated that TCR therapy was significantly more effective against malignant melanoma than LAK therapy. Allison et al. developed an immune checkpoint therapy by using an anti-CTLA-4 antibody that targets the T cell inhibitor molecule CTLA-4 [6]. Their study led to the clinical development of a drug called Ipilimumab, which resulted in improved survival in patients with malignant melanoma [7]. Immunotherapy is expected to make a significant contribution to the treatment of refractory malignant melanoma and other cancers by intentionally activating immune cells against tumor cells.

Mitogens and lectins are substances that activate immune cells [8]. Saito et al. reported that the sites that recognize sugar chains must be multivalent to realize lymphocyte activation [9]. These sites bind to glycoproteins called sugar chains on the surface of immune cells, and an activation signal is transmitted into the immune cells to induce proliferation [10]. However, lectin is a plant-derived protein and is thus antigenic and cytotoxic. As mentioned above, expensive proteins, such as antibody drugs and lectins, are used to activate immune cells, making immunotherapy difficult to apply clinically.

Our study was inspired by previous research on polymers with phenylboronic acid groups that have immune cell activity similar to lectins and focused on creating inexpensive excellent-handling ceramic materials to break through the current challenges. Boronic acid-containing polymers have the same activity as lectins for lymphocyte proliferation due to the interaction between the intracellular sugar chain and the OH group in B(OH)_3_ formed by the dissociation of the BO_2_ group in the phenylboronic acid group [11]. Furthermore, lectins called boronolectins [12] are expected to be applied to cancer immunotherapy because of their ability to induce cytotoxic cells [13,14].

Our study focused on boron-containing apatite (BAp; Ca_9.5+0.5x_{(PO_4_)_6−x_(BO_3_)_x_}{(BO_2_)_1−x_O_x_} (0 ≤ x ≤1)) ceramics. BAp was created via a solid solution of a functional group derived from boric acid into hydroxyapatite (HAp; Ca_10_(PO_4_)_6_(OH)_2_) [15]. BAp contains BO_2_ groups in its crystal structure, and a lower value of x indicates a greater amount of BO_2_ groups. Furthermore, BAp presents advantages, such as biocompatibility and simple sterilization, because it is bioceramic. In general, ceramics have better chemical corrosion resistance and thermal stability than polymers and metals. Therefore, the BAp ceramics in this study can be easily manipulated for sterilization and are useful as cell culture substrates. It is possible to activate immune cells from the same mechanism of action as lectins and phenylboronic acid polymers [10,11] by culturing them on BAp ceramics, using the BO_2_ groups as sites to recognize sugar chains [16]. Immune cells activated by this mechanism are induced to killer T cells [16].

Ceramics were prepared in the CaO-P_2_O_5_-SiO_2_-B_2_O_3_ system (CPSB ceramics) using the sol–gel process to synthesize materials as complex as BAp [17]. The sol–gel process allows the synthesis of highly homogeneous materials, starting from a solution and converting sol into gel through chemical reactions. Hydrolysis and polymerization reactions in solution can be used to obtain dried gels of fine particles, which can then be formed and sintered to produce ceramics with a microstructure of the desired chemical composition [18]. The CPSB ceramics synthesized by the sol–gel process were composed of BAp as the main crystalline phase and were, therefore, expected to have immune cell activity.

This study evaluated the cellular responsiveness of the CPSB ceramics prepared by the sol–gel process and verified their anti-tumor effect on malignant melanoma. First, immune cells were cultured on the CPSB ceramics, and the percentage of immune cells was evaluated by flow cytometry analysis to clarify the responsiveness of the CPSB ceramics. Next, the anti-tumor effect was verified by administering immune cells cultured on the CPSB ceramics to mice with malignant melanoma, measuring their tumor size, and histologically evaluating their tumor tissues.

Immunotherapy without side effects is an innovative medical treatment for cancer. However, common immunotherapy drugs are very expensive. Therefore, we propose the use of bioceramics that activate immune cells upon contact with surfaces. Bioceramics have been developed to replace some or all of the functions of the body and have been used as bone and tooth replacement materials [19]. One of the advantages of bioceramics is that they are rarely rejected by the immune system. However, if they can actively work on the immune system and activate immune cells such as killer T cells, they could offer a novel approach to “adoptive immunotherapy”, which is attracting attention as a treatment for cancer. Ultimately, it is expected to improve the quality of life of many people.

## 2. Results

### 2.1. Characterization of CPSB Ceramics

**Figure 1:**
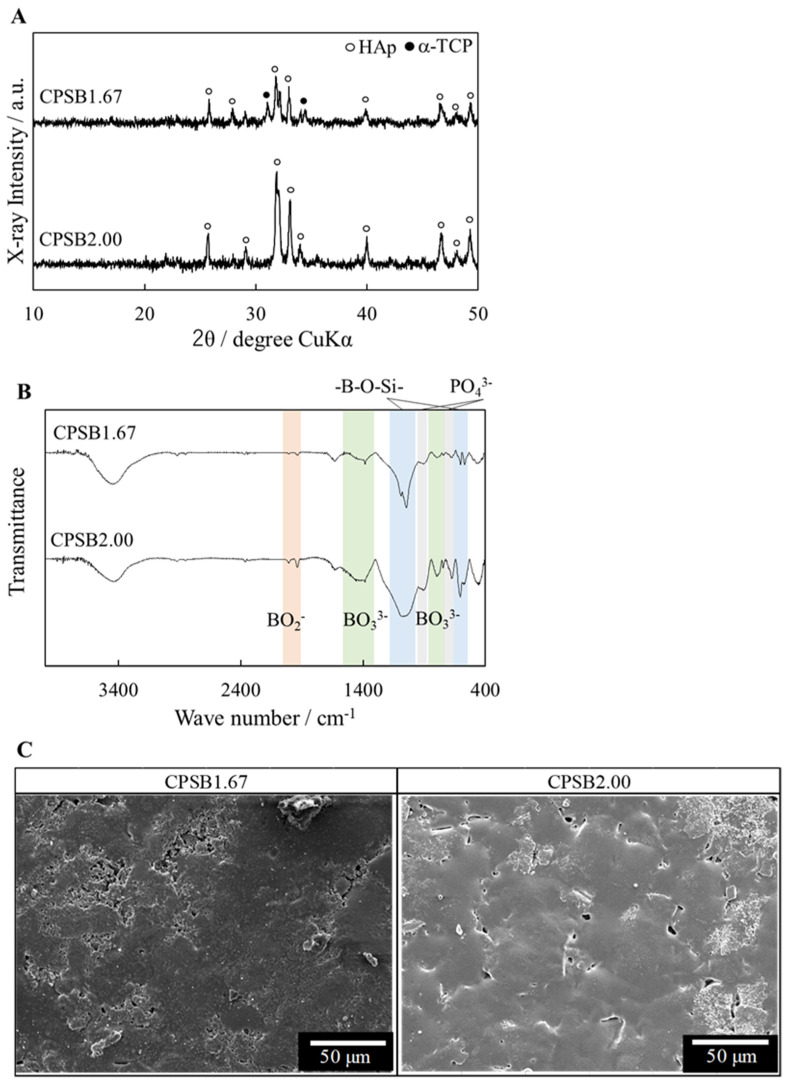
Characterization of CPSB ceramics. (**A**) XRD patterns (HAp: ICDD PDF#09–0432, α–TCP: ICDD PDF#09–0348), (**B**) FT–IR spectra, and (**C**) SEM images of CPSB ceramics.

Figure 1A displays the XRD patterns of the resulting CPSB ceramics. The XRD patterns indicate that the crystalline phases of CPSB1.67 comprise a small amount of α-tricalcium phosphate (α-Ca_3_(PO_4_)_2_; α-TCP: ICDD PDF#09-0348) and apatite phases (HAp: ICDD PDF#09-0432), while CPSB2.00 comprises a single apatite phase. Figure 1B displays the FT-IR spectrums of the resulting CPSB ceramics. FT-IR absorptions of BO_2_ and BO_3_ groups were detected in the CPSB ceramics, in addition to the PO_4_ groups. The absorptions of the BO_2_ group were detected at 2000 and 1940 cm^−1^, and those of the BO_3_ group were detected at 1600–1300 and 800–750 cm^−1^ [15]. These results indicate that BAp is the main crystalline phase of CPSB ceramics. Figure 1C presents SEM micrographs of the CPSB ceramic surfaces. These observations confirmed that pores were eliminated in all of the ceramics, and grain growth proceeded to densify the ceramics. HAp and CPSB ceramics are dense ceramics with a relative density of over 90% (Table 1). As the surface roughness of the HAp and BAp ceramics after polishing was almost constant (*R_α_* < 0.1 μm), the roughness was considered not to affect the cell culture.

### 2.2. Cellular Responses of Mice Spleen Cells to CPSB Ceramics

**Figure 2:**
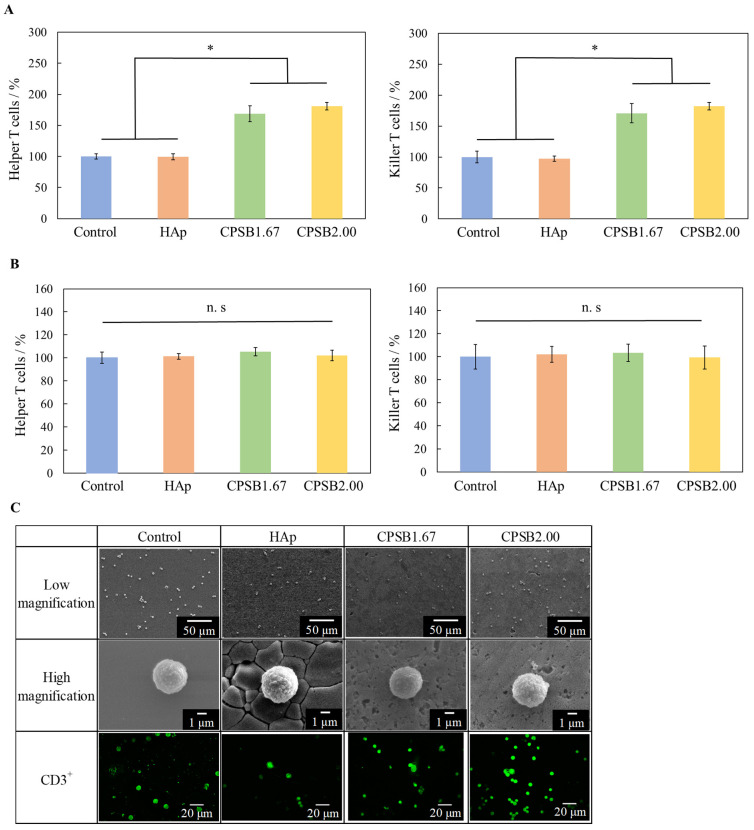
Cellular responses of mice spleen cells to CPSB ceramics. (**A**) Population of helper and killer T cells cultured on each substrate for one day. Data were analyzed by unpaired *t*-test (two-tailed). Error bar: standard deviation (S.D.); *n* = 4, * *p* < 0.01. (**B**) Population of helper and killer T cells cocultured without contact with each substrate for one day. Data are presented as mean ± S.D. (**C**) Morphological observation of spleen cells cultured on each substrate. Scale bars: 50 µm, 1 µm, and 20 µm. n.s: no significant difference.

The proportions of helper and killer T cells on each culture substrate were determined by flow cytometry analysis. Figure 2A presents the results, with the control set to 100%. The proportions of T cells on the CPSB ceramics (CPSB1.67 and CPSB2.00) were significantly higher than on the control and HAp ceramics. In addition, the proportions of helper and killer T cells cocultured without contact with each substrate using Transwell^®^ (Corning Inc., Corning, NY, USA) inserts were also determined by flow cytometry analysis with the control set to 100% (Figure 2B), and a difference was not observed in those proportions. The results indicate that liquid factors, such as ions released by the dissolution of ceramics, have little effect on the activation of immune cells. Therefore, the increased proportions of helper and killer T cells in splenocytes cultured on CPSB ceramics may be caused by the interaction between the ceramics and immune cells.

The morphology of spleen cells cultured on each sample was observed by SEM and immunofluorescence staining (Figure 2C). With immunofluorescence, green fluorescent-stained spherical T cells were observed on all culture substrates. On the SEM images, spleen cells were observed in contact with all culture substrates, and cells forming pseudopodia were also observed on the CPSB ceramics. Although most spleen cells do not adhere because they are floating, they may form pseudopodia and adhere when activated [20]. These results suggest that immune cells formed pseudopodia and adhered to their substrate when cultured on CPSB ceramics. Cell culture on CPSB ceramics affected the activation of immune cells.

### 2.3. Surface Zeta Potential of CPSB Ceramics

**Figure 3:**
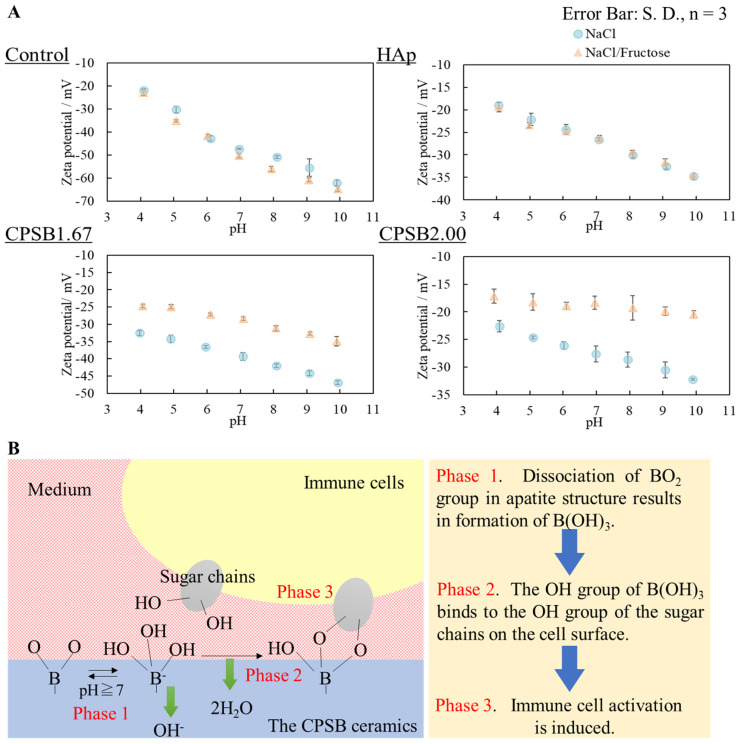
Verification of the interaction between CPSB ceramics and immune cell sugar chains. (**A**) Surface zeta potential of CPSB ceramics in NaCl/fructose solvent. (**B**) Model of the immune cell activation mechanism.

To clarify the activation mechanism of immune cells by CPSB ceramics, we built a model experiment in which fructose molecules were used as cellular sugar chains and indirectly verified the interaction between CPSB ceramics and immune cell sugar chains by measuring the surface zeta potential of the ceramics (Figure 3A). No difference in surface zeta potential was observed between the control and HAp due to the difference in solvents. On the other hand, the surface zeta potential of CPSB ceramics shifted more positively when the NaCl solution with fructose was used as a solvent. This observation may be explained by the surface zeta potential of CPSB ceramics shifting negatively due to the dissociation of BO_2_ groups on their surface and the interaction of fructose with the BO_2_ groups, causing the negatively charged surface zeta potential to shift positively.

### 2.4. Anti-Tumor Effects In Vivo

**Figure 4:**
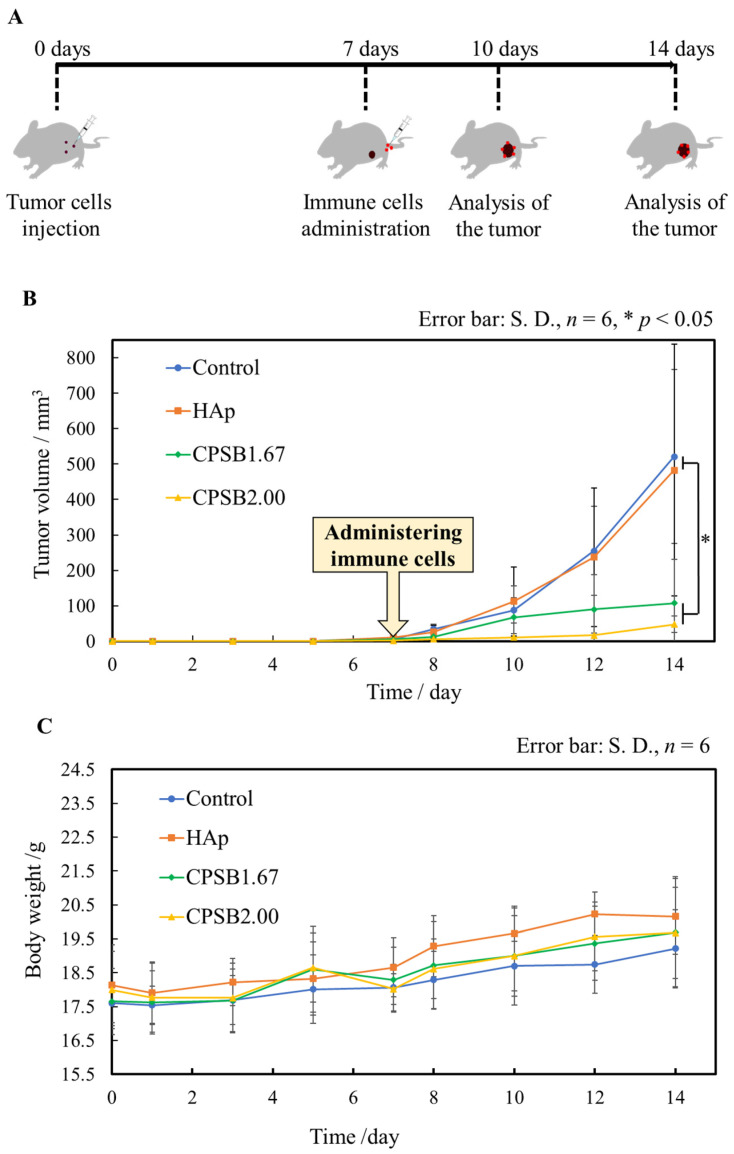
Verification of anti-tumor effects in vivo. (**A**) Flow chart of the in vivo experiment. (**B**) Tumor volume of tumor-bearing mice treated with immune cells cultured on each substrate. Data were analyzed by unpaired *t*-test (two-tailed). Error bar: S.D., *n* = 6, * *p* < 0.05. (**C**) Body weight of tumor-bearing mice treated with immune cells cultured on each substrate. Data are presented as mean ± S.D.

Melanoma tumor-bearing mice were obtained, and the immune cells activated on the CPSB ceramics were injected into the tail vein of the mice to verify their anti-tumor effects. The mice were euthanized three and seven days after injection for tumor analysis (Figure 4A). Figure 4B displays the tumor volume of tumor-bearing mice treated with immune cells cultured on each culture substrate. Tumors were found to form around seven days after administrating B16-F10 OVA cells to all mice. After that, immune cells cultured on each culture substrate for 1 day were administered to the tumor-bearing mice. Compared with the control and HAp, immune cells cultured on the CPSB ceramics significantly inhibited tumor growth (Figure 4C). All treated mice displayed a gradual increase in body weight, regardless of the tumor volume. These results indicate that the treatment can be performed without causing any health problems due to the administration of immune cells.

**Figure 5:**
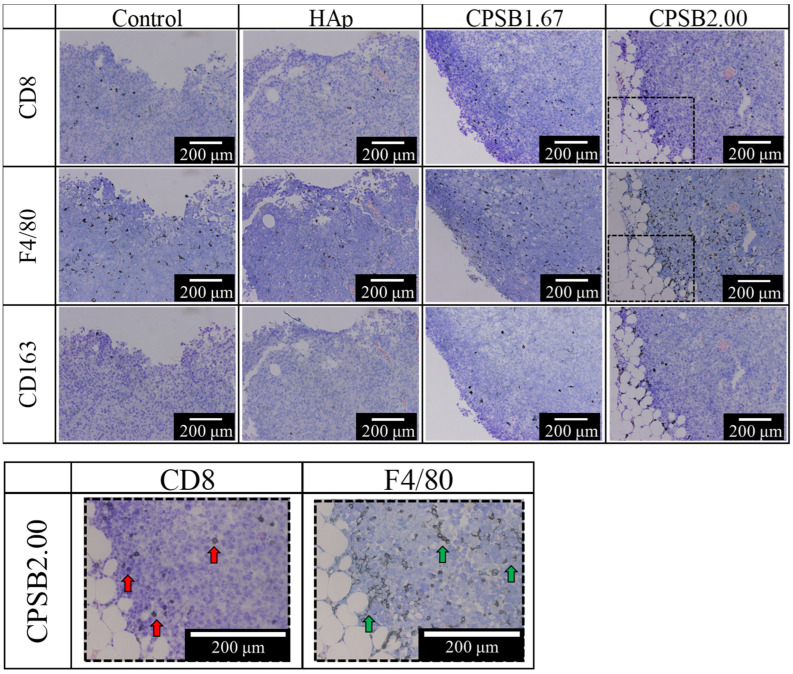
Staining images ten days after treatment of tumor-bearing mice with immune cells cultured on each substrate. These images allowed us to observe the behavior of immune cells three days after administrating immune cells. The immune cells stained dark brown by CD8 (marker of killer T cells), F4/80 (marker of M1 and M2 macrophages), and CD163 (marker of M2 macrophages) antibodies were considered positive. The larger versions of the black frame area are shown in the image below. Red arrows, CD8 antibody-stained killer T cells; green arrows, F4/80 antibody-stained macrophages.

Figure 5 presents images of tumor tissue stained with CD8 (marker of killer T cells), F4/80 (marker of M1 and M2 macrophages), and CD163 (marker of M2 macrophages) antibodies ten days after administrating B16-F10 OVA cells. The behavior of immune cells was observed three days after administration. The immune cells stained dark brown by CD8, F4/80, and CD163 antibodies were considered positive. CD8^+^ T cells observed with CD8 antibody staining are thought to have infiltrated the outside of the tumor tissue. Compared with the control and HAp surfaces, more CD8^+^ T cells were found to have infiltrated the tumor tissue by administrating immune cells cultured on the CPSB ceramics. In fact, CD3^+^ CD8^+^ T cells can infiltrate the tumor tissue three days after tail vein injection [21]. These results suggest that CD3^+^ CD8^+^ T cells activated on the CPSB ceramics infiltrate tumor tissues three days after administration and begin attacking tumor cells, expressing effective anti-tumor effects. Next, macrophages near the outside of the tumor tissue were observed from staining with F4/80 and CD163 antibodies. Macrophages move (skewing/polarization) between two states, M1 and M2, depending on changes in the tissue microenvironment [22]. M1 macrophages produce many inflammatory cytokines and eliminate foreign substances from the body, whereas M2 macrophages have anti-inflammatory properties, suppress anti-tumor immunity, produce various angiogenic factors, and induce neovascularization to create a favorable microenvironment for cancer cell growth [23]. M1 macrophages phagocytosing dead tumor cells activate CD3^+^ CD8^+^ cells by presenting cancer cell antigens to them and provide a favorable microenvironment for anti-tumor immunity [24]. As a result, M1 macrophages play a role in enhancing anti-tumor effects. The F4/80 antibody stains M1 and M2 macrophages, while the CD163 antibody stains only M2 macrophages, indicating that CD163^+^ cells are M2 macrophages while F4/80^+^ and CD163^−^ cells are M1 macrophages. From staining with F4/80 and CD163 antibodies, more M1 macrophages infiltrating the tumor tissue were observed by administrating immune cells cultured on the CPSB ceramics than on the control and HAp surfaces. These results indicate that administrating immune cells cultured on CPSB ceramics inhibits tumor cell proliferation from three days after injection.

**Figure 6:**
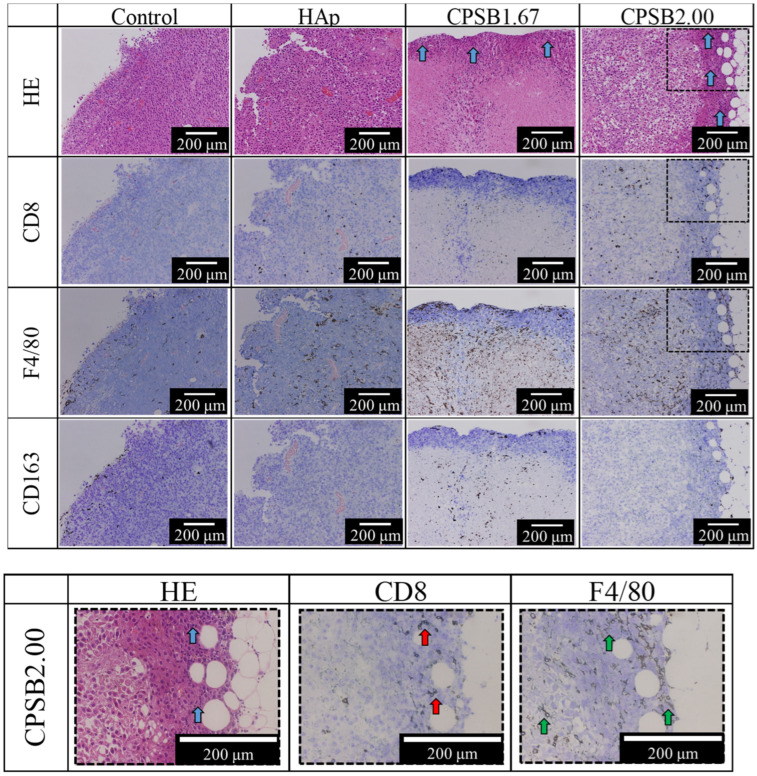
Staining images 14 days after treatment of tumor-bearing mice with immune cells cultured on each substrate. The behavior of tumor and immune cells seven days after administrating immune cells was observed in HE staining and immunohistochemical staining images with CD8, F4/80, and CD163 antibodies. The larger versions of the black frame area are shown in the image below. Red arrows, CD8 antibody-stained killer T cells; green arrows, F4/80 antibody-stained macrophages; blue arrows, HE staining images showing dead tumor cells.

Figure 6 presents staining images of tumor tissues taken 14 days after administrating B16-F10 OVA cells using HE and immunohistochemical staining with CD8, F4/80, and CD163 antibodies. First, anti-tumor effects were evaluated by observing the appearance of living and dead tumor cells near the outside of the tumor tissue by HE staining. As a method of determining whether tumor cells are alive or dead, cells with large nuclei, a light purple color, and large cytoplasmic areas are considered living tumor cells, while cells with smaller nuclei and a darker appearance are considered dead tumor cells. Compared with the control and HAp surfaces, administrating immune cells cultured on the CPSB ceramics confirmed the presence of dead tumor cells near the outside of the tumor (Figure 6, blue arrows).

Next, anti-tumor effects were evaluated by observing the appearance of killer T cells that might have infiltrated the tumor tissue from staining with the CD8 antibody. Compared with the control and HAp surfaces, administrating immune cells cultured on the CPSB ceramics confirmed the presence of killer T cells near the dead tumor cells outside of the tumor. These results suggest that the killer T cells activated on the CPSB ceramics exerted an effective anti-tumor activity by attacking tumor cells.

Finally, anti-tumor effects were evaluated by observing the appearance of M1 macrophages that might have infiltrated the tumor tissue from staining images with F4/80 and CD163 antibodies. Compared with the control and HAp surfaces, administrating immune cells cultured on the CPSB ceramics confirmed the presence of more M1 macrophages in the tumor tissue. These results indicate that administrating immune cells cultured on CPSB ceramics provided a favorable microenvironment for anti-tumor immunity and inhibited tumor cell proliferation until seven days after injection. After ten days, killer T cells activated on the CPSB ceramics infiltrated the tumor tissue and started attacking tumor cells. After 14 days, a lot of killer T cells and M1 macrophages infiltrated and provided a favorable microenvironment for anti-tumor immunity. Furthermore, the presence of dead tumor cells in the vicinity of killer T cells was confirmed, confirming that administrating activated immune cells on CPSB ceramics exerted anti-tumor effects. In summary, the administration of killer T cells activated on CPSB ceramics to mice significantly suppressed tumor growth as killer T cells attacked tumor cells and M1 macrophages created favorable environments for anti-tumor effects.

## 3. Discussion

The XRD patterns indicate that the crystalline phases of CPSB1.67 comprise a small amount of α-TCP and apatite phases, while CPSB2.00 comprises a single apatite phase. These results suggest that apatite is more likely to be formed when the composition of the Ca/P ratio is higher than the theoretical value of 1.67 for HAp [17]. FT-IR absorptions of BO_2_ and BO_3_ groups were detected in the CPSB ceramics in addition to the PO_4_ groups. These results indicate that BAp was the main crystalline phase of the CPSB ceramics. The lattice constant (*c*-axis) of the CPSB ceramics increased compared with the lattice constant of stoichiometric HAp (ICDD PDF#09-0432). The B atom was linearly coordinated at the OH site in the apatite structure to form a BO_2_ group, and the lattice constant was considered to expand to the c-axis due to the substitution of the BO_2_ group with a larger molecular size compared with the OH group [16,25].

Next, we explain the cellular responses of mice spleen cells to CPSB ceramics. The results shown in Figure 2B indicate that when the immune cells cocultured without contact with each substrate liquid factors, such as ions released by the dissolution of ceramics, it had little effect on the activation of immune cells. Meanwhile, the results shown in Figure 2A,C indicate that when the immune cells were cultured in the presence of ceramics, stimulation by the adhesion of cells to the ceramics may affect their activity and maturation. Therefore, the increased proportions of helper and killer T cells in splenocytes cultured on CPSB ceramics may be caused by the interaction between the ceramics and immune cells. The cellular responses of spleen cells to CPSB2.00 was the best; therefore, it was considered that the cells were activated by culturing on a material with a large amount of BO_2_ groups [16]. The tendency was significant in the CPSB2.00 because it had many BO_2_ groups, as shown by the FT-IR absorption of its general formula, and was a single BAp phase. Furthermore, to clarify the activation mechanism of immune cells by CPSB ceramics, we indirectly verified the interaction between CPSB ceramics and immune cell sugar chains (Figure 3A). These results revealed the mechanism of immune cell activation (Figure 3B). First, the dissociation of the BO_2_ group in apatite structure resulted in the formation of B(OH)_3_ (phase 1). Next, the OH group of B(OH)_3_ bound to the OH group of the sugar chains on the cell surface (phase 2). Finally, immune cell activation was induced. Therefore, BO_2_ groups were thought to dissociate on the surface of the CPSB ceramics in the solvent and interact with cellular sugar chains. When immune cells were cultured on the CPSB ceramics, they were activated by the interaction of BO_2_ groups dissociated from the material surface and the sugar chains of immune cells. The activation of immune cells by the interaction of BO_2_ groups on the surface with sugar chains of immune cells is the same mechanism of action as that of phenylboronic acid polymers [15], as demonstrated by the zeta potential measurements of the ceramic surfaces in Figure 3A.

**Figure 7:**
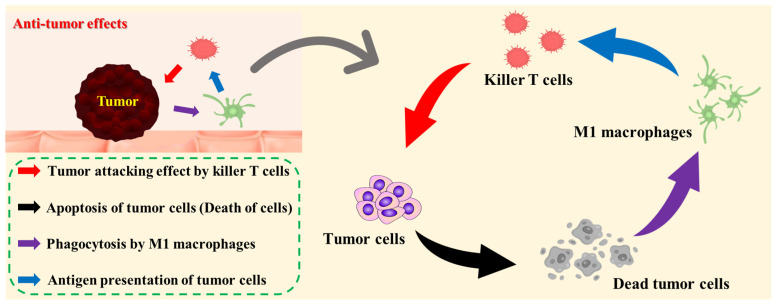
Model diagram of adoptive immunotherapy for tumor-bearing mice and mechanism of its anti-tumor effects.

Finally, anti-tumor effects are discussed. Figure 7 presents a model diagram of adoptive immunotherapy for tumor-bearing mice and the mechanism of their anti-tumor activity. In this study, we developed adoptive immunotherapy in which immune cells cultured and activated on the CPSB ceramics were administered to tumor-bearing mice. Compared with the control and HAp surfaces, administrating immune cells cultured on the CPSB ceramics significantly inhibited tumor growth in tumor-bearing mice. In addition, the histological evaluation of CPSB ceramics indicated the presence of many dead cells, killer T cells, and M1 macrophages in cancer cell tissues. Based on these results, the following mechanism was deduced. First, killer T cells activated on CPSB ceramics attacked tumor cells and, then, M1 macrophages phagocytosed dead tumor cells. M1 macrophages presented cancer cell antigens to killer T cells, which strengthened the attack of killer T cells. In this study, we demonstrated that the effective activation of killer T cells working by this mechanism on CPSB ceramics produced remarkable anti-tumor effects.

## 4. Materials and Methods

### 4.1. Fabrication of CPSB Ceramics by Sol–Gel Process and Their Characterization

Two kinds of CPSB ceramics with Ca/P ratios of 1.67 and 2.00 were fabricated by the sol–gel process [17]. The abbreviation “CPSB1.67” was used to indicate the Ca/P ratio of the oxide composition. Sample solutions were prepared by mixing Ca(NO_3_)_2_·4H_2_O (FUJIFILM Wako Pure Chemical Co., Ltd., Osaka, Japan, reagent special grade), (NH_4_)_2_HPO_4_ (FUJIFILM Wako Pure Chemical Co., Ltd., Osaka, Japan, reagent special grade), Si(OC_2_H_5_)_4_ (FUJIFILM Wako Pure Chemical Co., Ltd., Osaka, Japan, reagent special grade), H_3_BO_3_ (FUJIFILM Wako Pure Chemical Co., Ltd., Osaka, Japan, reagent special grade), C_2_H_5_OH (FUJIFILM Wako Pure Chemical Co., Ltd., Osaka, Japan, reagent special grade), and HNO_3_ (FUJIFILM Wako Pure Chemical Co., Ltd., Osaka, Japan, reagent special grade, 60 mass%) (Table 2). Si(OC_2_H_5_)_4_ was dissolved into ethanol (1:10), and the mixture was stirred vigorously. After the nitric acid solution including Ca(NO_3_)_2_ 4H_2_O, (NH_4_)_2_HPO_4_, and H_3_BO_3_ was added to the ethanol solution, the mixed solutions were stirred for one additional hour. The molar amounts of H_2_O were ten times as large as those required for the hydrolysis of Si(OC_2_H_5_)_4_. Although the pH conditions for each process were not measured, HNO_3_ was added to the solution to make the solution acidic and hydrolyzed Si(OC_2_H_5_)_4_. The solutions were allowed to stand for ten days at room temperature and then gelatinized. The obtained gels were dried at various temperatures, e.g., 80, 100, 150, and 200 °C for 8 h each. The dried gel powders were heated up to 200 °C at 10 °C min^−1^ and from 200 to 500 °C at 1 °C·min^−1^; they were then held at 500 °C for 1 h. The calcined powders were ground to pass through 150 µm sieves. Approximately 1 g of the calcined powder was uniaxially pressed at 100 MPa and heated at 1100 °C for 1 h. The resulting ceramics were surface-polished and ultrasonically cleaned to obtain CPSB ceramics. The surface roughness after polishing was consistent at 0.1 µm or less.

For the characterization of CPSB ceramics, the crystalline phases of the obtained ceramics were identified with an X-ray powder diffractometer (XRD) using CuKα radiation (Miniflex, Rigaku, Tokyo, Japan, counter cathode: Cu; accelerating voltage: 30 kV; current: 15 mA; scanning range 2θ: 10°~50°; scanning speed: 2°·min^−1^; scanning step: 0.02°).The crystalline phases were checked referring to the International Centre for Diffraction Data (ICDD) cards. Fourier-transformed infrared (FT-IR) measurements were conducted using the KBr method (IR Prestige-21, Shimadzu, Kyoto, Japan, measurement mode: %transmittance; appotize function: Happ–Genzel; cumulative number: 40 times; number of scans: 20 times; resolution: 4 cm^−1^; scanning speed: 2.5 mm·s^−1^; measurement range: 400~4000 cm^−1^). The microstructure was observed using a scanning electron microscope (SEM; JSM-6390LA, JEOL, Tokyo, Japan, accelerating voltage: 10 kV; vacuum mode: HV; signal: SEI; Z axis: 10 mm (9~11 mm); spot size: 30). Samples were attached to an aluminum sample stand using carbon double-sided tape, and Pt was deposited using AUTO FINE COATER (JFC-1600, JEOL, Tokyo, Japan, coating time: 70 s; sputtering current: 30 mA) as a sample for observation. The relative density (%; [bulk density, g·cm^−3^]/[true density, g·cm^−3^] × 100) was calculated using the sintered compacts; the true density was psychometrically determined using ethanol as an immersing solution; and the bulk density was calculated from the dimensions and weight. The experiment with n = 3 samples was repeated 3 times to obtain the results. The resulting ceramics had a diameter of about 15 mm and a thickness of about 2.0 mm. The lattice constant of the CPSB ceramics was measured by XRD (Ultima IV, Rigaku, Tokyo, Japan, cathode: Cu; filter: fully automatic monochromator; accelerating voltage: 40 kV; tube current: 40 mA; scanning range 2θ: 10°~50°; scanning speed: 0.1°·min^−1^; scanning step: 0.02°). Lattice constants were refined from the least-squares method using the control software (PDXL 2, ver.2.1.3.6) provided with the XRD instrument. Silicon powder (National Institute of Standards and Technology, standard substance 640 d) was used as a standard substance. After measurement, a comparison of the lattice constant was performed using the ICDD card of HAp. The resulting HAp and CPSB ceramics were surface-polished, ultrasonically cleaned, and subjected to dry-heat sterilization for cell culture.

### 4.2. Mice and Cell Culture

All animal treatments were approved by the Animal Research Committee of Meiji University (Approval No.: MUIACUC 2020-11). Spleen cells were obtained from 7–12-week-old female C57BL/6N mice. Spleens were removed from the mice, and spleen cells were isolated using a nylon mesh sheet of 67 μm in diameter. Next, red blood cells were removed from the spleen cells with ammonium chloride potassium (ACK) lysis buffer and washed. The ACK lysis buffer was prepared with ammonium chloride (NH_4_Cl, FUJIFILM Wako Pure Chemical Co., Ltd., Osaka, Japan), potassium bicarbonate (KHCO_3_, FUJIFILM Wako Pure Chemical Co., Ltd., Osaka, Japan), and ethylenediamine-N,N,N′,N′-tetraacetic acid disodium salt dihydrate (FUJIFILM Wako Pure Chemical Co., Ltd., Osaka, Japan). Spleen cells were cultured in a RPMI 1640 medium (Nissui Pharmaceutical Co., Ltd., Tokyo, Japan) supplemented with 10% fetal bovine serum, 100 U·cm^−3^ penicillin, 100 μg·cm^−3^ streptomycin, and 50 mmol·dm^−3^ 2-mercaptoethanol. Splenocytes (1.0 × 10^6^ cells·cm^−3^) were seeded on the above HAp or CPSB ceramics or onto a polystyrene plate (24-well polystyrene plate) as a control, then cultured for 1 day at 37 °C in a humidified atmosphere with 5% CO_2_. HAp ceramics were fabricated by uniaxially pressing 1.0 g of HAp-100 powder (Taihei Chemical Industrial Co., Ltd., Osaka, Japan) at 50 MPa and burning at 1200 °C for 5 h. Splenocytes were cultured in a non-contact environment with ceramics using Transwell^®^ inserts to clarify the interaction of the cells with the ceramics. An amount of 1.0 cm^3^ of splenocyte suspension (1.0 × 10^6^ cells·cm^−3^) was seeded onto 12-well polystyrene plates. Each ceramic (0.3 g of calcined powder was molded using a 10.0 mm diameter molding device, and samples were prepared as in Section 4.1) was placed in a Transwell^®^ insert, and 0.8 cm^3^ of medium was added. Spleen cells were cultured in an RPMI 1640 medium for 1 day at 37 °C in a humidified atmosphere with 5% CO_2_.

### 4.3. Flow Cytometry

One day after cultivation, the proportion of helper T (CD3^+^CD4^+^) and killer T (CD3^+^CD8^+^) cells was estimated using a flow cytometer (Attune acoustic focusing cytometer; Thermo Fisher Scientific, Waltham, MA, USA). Splenocytes were suspended in 2 cm^3^ of fluorescence-activated cell-sorting (FACS) buffer. Samples were washed with 2 cm^3^ FACS buffer and resuspended in 1 cm^3^ FACS buffer. The abovementioned cell suspension (1 cm^3^) was treated with 110 mm^3^ of a mixture containing 15 mm^3^ of antibody. CD3 (FITC hamster anti-mouse CD3e, clone 145-2C11; BD Pharmingen, San Diego, CA, USA), CD4 (PE rat anti-mouse CD4/L3T4a, clone GK1.5; BECKMAN COULTER, Brea, CA, USA), and CD8 (Pacific Blue rat anti-mouse CD8a, clone 5H10; Invitrogen, Waltham, MA, USA) antibodies were used in this experiment. CD3 is a pan T cell marker, and CD4 or CD8 are helper subset or cytotoxic subset markers for T cells, respectively. Cells were incubated at 4 °C for 15 min and washed with 2 cm^3^ FACS buffer. Data analysis was performed using the Attune cytometric software (Attune Cytometric Software, ver. 1.2.5; Applied Biosystems, Waltham, MA, USA). Gates were set appropriately with unstimulated controls. Voltages were determined from unstained controls.

### 4.4. Morphological Observation of Cells

Spleen cells cultured for one day were observed by immunofluorescence staining and SEM. For SEM, cells were washed three times with PBS and fixed in a 10% glutaraldehyde solution (FUJIFILM Wako Pure Chemical Co., Ltd., Osaka, Japan) at 4 °C. Next, cells were washed five times with sterilized water, freeze-dried, and observed. For immunofluorescence staining, cells were washed three times with PBS and fixed in 4% paraformaldehyde phosphate (FUJIFILM Wako Pure Chemical Co., Ltd., Osaka, Japan) at 4 °C. Cells were then washed three times with PBS and immersed in 0.5 cm^3^ of 0.11% TritonX-100 (Merck KGaA, Darmstadt, Germany) for 10 min at room temperature. Next, they were washed three times with PBS and blocked by immersion in 0.5 cm^3^ of 3% BSA (FUJIFILM Wako Pure Chemical Co., Ltd., Osaka, Japan) for 1 h. After three more washes with PBS, cells were stained with the antibody solution for 1 h at room temperature and washed three times again with PBS before observation. The antibody used for staining was the hamster anti-mouse CD3 antibody (clone 145-2C11, Isotype IgG, Bio-Rad Laboratories, Hercules, CA, USA), which made the T cells fluorescent green.

### 4.5. Evaluation of the Interaction between Ceramic Surfaces and the Intracellular Sugar Chain by Model Experiments

We constructed a model experiment in which fructose molecules were used as cellular sugar chains and indirectly verified the interaction between CPSB ceramics and immune cellular sugar chains by determining the surface zeta potential of CPSB ceramics. In this experiment, the surface zeta potential of ceramics was measured using the laser Doppler method using a Zeta-potential analyzer (ELSZ-2, Otsuka Electronics, Osaka, Japan). Polystyrene latex (particle size about 500 nm, Otsuka Electronics) coated with hydroxypropyl cellulose was used as the monitor particle. Monitor particles were dispersed in two solvents: (i) 0.01 mol·dm^−3^ NaCl solution and (ii) a solution with 0.20 mol·dm^−3^ fructose and 0.01 mol·dm^−3^ NaCl. The specimens with the dimensions of 24.5 mm (long) × 11.0 mm (wide) × 2.0 mm (thick) were compressed at 50 MPa and heated at 1100 °C for 1 h. The surface potential of glass slides was measured as a control, and the surface potential of HAp ceramics with the abovementioned dimensions was measured for comparison with CPSB ceramics.

### 4.6. Tumor Treatment Study in Mice

In this experiment, melanoma cells (B16-F10-OVA cells) expressing ovalbumin (OVA) antigen were injected subcutaneously into the left abdomen of mice (C57BL/6N, female, six weeks old) to establish tumors. The number of B16-F10-OVA cells was 5 × 10^5^ cells/50 mm^3^ for each mouse. Independently, CD3^+^CD8^+^ T cells expressing specific receptors for OVA antigens (harvested and isolated from OT-1 transgenic mice) were cultured on CPSB ceramics. B16-F10 cells express a molecule called PD-L1 (programmed death-ligand 1) on their surface, which binds to a molecule called PD-1 (programmed death-1) expressed on T cells and acquires the ability to escape T cell attack [26]. Therefore, B16-F10-OVA cells expressing OVA antigens were established in mice as tumors, and CD3^+^CD8^+^ T cells expressing specific receptors for OVA antigens were administered to tumor-bearing mice to avoid the immune escape of tumor cells [27]. CD3^+^CD8^+^ T cells (2.0 × 10^7^ cells·cm^−3^) were cultured on the CPSB and HAp ceramics for 1 day at 37 °C in a humidified atmosphere with 5% CO_2_. The thus-activated CD3^+^CD8^+^ T cells (1 × 10^6^ cells·50 mm^−3^) were collected and injected into the tail vein of the tumor-bearing mice. CD3^+^CD8^+^ T cells were injected seven days after the administration of B16-F10-OVA. The tumor volume and the body weight of tumor-bearing mice were measured every two days. The tumor volume was calculated as follows: tumor volume = 1/2 × (major axis) × (minor axis)^2^.

### 4.7. Histological Evaluation

Tumor tissues were removed from tumor-bearing mice 10 and 14 days after administrating B16-F10-OVA cells, washed three times with 1 cm^3^ of PBS, and immersed in 2 cm^3^ of PBS for 24 h. The tumor tissue was washed three more times with 1 cm^3^ of PBS and fixed with 2 cm^3^ of 4% paraformaldehyde/PBS at 4 °C. After three more washes with 1 cm^3^ of PBS and immersion in PBS, the tumor tissues were stained by Morpho Technology Co. (Sapporo, Japan). Histological evaluation was performed by hematoxylin–eosin (HE) and immunohistochemical staining with CD8, F4/80, and CD163 antibodies. Immunohistochemical staining was performed after Giemsa staining. HE staining stained the nuclei of the tumor cells in dark blue/purple and the cytoplasm in pale red. On the other hand, Giemsa staining stained the nuclei of the tumor cells in dark blue and the cytoplasm in sky blue. CD8, F4/80, and CD163 antibodies, respectively, stained killer T cells, macrophages (M1 and M2), and M2 macrophages in dark brown.

## 5. Conclusions

The present CPSB ceramics were created for novel cancer immunotherapy. Immune cells were cultured on the CPSB ceramics to evaluate immune cell responsiveness. Immune cells activated on CPSB ceramics were administered to melanoma tumor-bearing mice to verify their anti-tumor effects. The results of material characterization indicated that BAp was the main crystalline phase of the CPSB ceramics. Flow cytometry analysis and cell morphology observations revealed that CPSB ceramics activated immune cells. In addition, model experiments using fructose molecules as cellular sugar chains clarified that the activation of immune cells was due to the interaction between dissociated BO_2_ groups in CPSB ceramics and the sugar chains of immune cells. Administrating immune cells cultured on CPSB ceramics to malignant melanoma tumor-bearing mice significantly inhibited tumor growth. Compared with the results of immunocytes cultured on HAp ceramics and administered to malignant melanoma tumor-bearing mice, the tumor reduction ratios were 22.17% for CPSB1.67 and 9.856% for CPSB2.00, indicating the remarkable anti-tumor effects of CPSB ceramics. Furthermore, the histological evaluation of the tumors indicated that immune cells cultured on CPSB ceramics effectively inhibited tumor growth in mouse tumor tissues. Our research brings forth CPSB ceramics as a valuable biomaterial for immunotherapy without relying on typical drugs.

## 6. Patents

Japan patent: Tokugan 2022-117028.

## Figures and Tables

**Table 1:** Material properties of CPSB ceramics.

Sample Name	Relative Density/%	Lattice Constant/nm		Surface Roughness/μm
		*a*(*b*) Axis	*c* Axis	
HAp	96.6	0.942	0.688	0.067
CPSB1.67	90.3	0.943	0.693	0.064
CPSB2.00	93.3	0.941	0.695	0.057

**Table 2:** Nominal composition of the CPSB ceramics.

Sample Name	CaO/mol%	P_2_O_5_/mol%	SiO_2_/mol%	B_2_O_3_/mol%	Ca/P Molar Ratio
CPSB1.67	30.78	9.22	50.00	10.00	1.67
CPSB2.00	32.00	8.00	50.00	10.00	2.00

## Data Availability

The data that support the findings of this study are available from the corresponding author (M.A.) upon reasonable request.
